# Reliable allele detection using SNP-based PCR primers containing Locked Nucleic Acid: application in genetic mapping

**DOI:** 10.1186/1746-4811-3-2

**Published:** 2007-02-07

**Authors:** Joy Nakitandwe, Friederike Trognitz, Bodo Trognitz

**Affiliations:** 1 Bioresources Department, Austrian Research Centers GmbH – ARC, A-2444, Seibersdorf, Austria

## Abstract

**Background:**

The diploid, *Solanum caripense*, a wild relative of potato and tomato, possesses valuable resistance to potato late blight and we are interested in the genetic base of this resistance. Due to extremely low levels of genetic variation within the *S. caripense *genome it proved impossible to generate a dense genetic map and to assign individual Solanum chromosomes through the use of conventional chromosome-specific SSR, RFLP, AFLP, as well as gene- or locus-specific markers. The ease of detection of DNA polymorphisms depends on both frequency and form of sequence variation. The narrow genetic background of close relatives and inbreds complicates the detection of persisting, reduced polymorphism and is a challenge to the development of reliable molecular markers. Nonetheless, monomorphic DNA fragments representing not directly usable conventional markers can contain considerable variation at the level of single nucleotide polymorphisms (SNPs). This can be used for the design of allele-specific molecular markers. The reproducible detection of allele-specific markers based on SNPs has been a technical challenge.

**Results:**

We present a fast and cost-effective protocol for the detection of allele-specific SNPs by applying Sequence Polymorphism-Derived (SPD) markers. These markers proved highly efficient for fingerprinting of individuals possessing a homogeneous genetic background. SPD markers are obtained from within non-informative, conventional molecular marker fragments that are screened for SNPs to design allele-specific PCR primers. The method makes use of primers containing a single, 3'-terminal Locked Nucleic Acid (LNA) base. We demonstrate the applicability of the technique by successful genetic mapping of allele-specific SNP markers derived from monomorphic Conserved Ortholog Set II (COSII) markers mapped to Solanum chromosomes, in *S. caripense*. By using SPD markers it was possible for the first time to map the *S. caripense *alleles of 16 chromosome-specific COSII markers and to assign eight of the twelve linkage groups to consensus Solanum chromosomes.

**Conclusion:**

The method based on individual allelic variants allows for a level-of-magnitude higher resolution of genetic variation than conventional marker techniques. We show that the majority of monomorphic molecular marker fragments from organisms with reduced heterozygosity levels still contain SNPs that are sufficient to trace individual alleles.

## Background

Single nucleotide polymorphisms (SNPs) represent the most common variations across a genome [1], they occur at a frequency of about one SNP in 1000 nucleotides in genomic DNA [2] and they can be used to directly detect alleles responsible for a trait of interest. SNPs have several uses in genetics including the detection of alleles associated with heritable human diseases [3] and inferences of population history [4]. SNPs can also be used for fingerprinting and the generation of genetic maps, although these techniques frequently employ marker techniques that do not require any knowledge of genetic sequence. In general, popular techniques employ markers based on length differences, such as SSR [5,6], on alterations within restriction sites of DNA cutting enzymes, such as RFLP [7,8], AFLP [9], and CAPS [10], and on short polymorphic sequences, such as gene- and allele-specific markers (SCAR [11] and DALP [12]). Combinations of these principles often are applied to increase the number of useful polymorphisms detected in a limited number of steps. While having the advantage of being applicable at the species level and also in less-studied genomes, the common drawback of all these marker technologies is their dependence on the distribution and frequency of redundant, global features across a genome. A global marker technique that relies on the recognition site of a specific restriction enzyme can maximally detect all the corresponding restriction sites within a genome. In contrast, every SNP in context with its surrounding genomic sequence is unique. SNPs can mark functionally important allelic differences, and SNPs that flag individual alleles of known genes have been used widely as molecular markers. For example, in plants, the hypervariable self-incompatibility locus has been studied by applying allele-specific (AS) PCR primers (see [13]). In another example, alleles of the MDM2 locus associated with human breast cancer were detected by SNP genotyping [14]. The enhanced reproducibility of SNP genotyping using PCR primers consisting of Locked Nucleic Acid (LNA) to detect human disease-associated alleles has been demonstrated [15,16], and Latorra *et al*. [17] described the improved specificity of AS PCR primers containing 3'-LNA residues, compared to native DNA primers.

An advanced approach to genotyping comprises the exploitation of SNPs that flag individual alleles of conventional markers. It combines the advantages of markers that tag single or multiple loci and genome-wide, global features, and the property of SNP markers to discriminate alleles at the level of individual organisms.

We have developed a protocol for genetic fingerprinting of diploid individuals using SNPs from within monomorphic and therefore non-informative, conventional markers. Direct sequencing of conventional marker fragments led to the detection of allele-specific SNPs, and AS PCR was primed at these SNPs. To maximise the selectivity of the SNP-specific PCR, 3'-LNA-modified primers proved most successful. This time- and cost-efficient approach was used to map informative genetic loci in the highly homogeneous genetic background of *S. caripense *after conventional marker techniques had failed. *S. caripense *is a relative of potato and tomato, and the consensus potato and tomato maps [18] exemplify the close relationship among species within the genus Solanum. However, the goal of assigning the anonymous linkage groups on an initial *S. caripense *map (F. Trognitz and J. Nakitandwe, unpublished) by applying conventional SSR, CAPS, SCAR and Conserved Ortholog Sequence II (COSII; see Methods) markers of known position on the Solanum genetic maps could not be achieved. As an example, only six of 25 SSR markers previously mapped in potato and tomato amplified DNA from *S. caripense*, two of these were polymorphic but they had fragment sizes different from the markers previously mapped in potato and tomato and therefore could not be used to assign *S. caripense *chromosomes. The low level of heterozygosity in our *S. caripense *mapping population was testified by the small average number of only 10 markers per AFLP primer combination (318 polymorphic AFLP fragments obtained from genotyping with 31 primer pairs, results not shown). In contrast, Li *et al*. [19] obtained an average of 54 polymorphic AFLP bands per primer-restrictase combination in potato while Haanstra *et al*. [20] reported 49 in tomato. The overall limited degree of marker polymorphism obtained on our *S. caripense *genotypes indicates extreme levels of homogeneity and presence of minimum allelic diversity. This is surprising as these genotypes express strict self-incompatibility (SI, B. Trognitz, unpublished results). SI favours outbreeding and heterozygosity in large populations. In small, isolated populations of outbreeders, heterozygosity could be reduced to those parts of a genome that are closely linked to SI loci and our material may represent the latter type of populations [compare [21]].

Due to the failure to detect sufficient polymorphism in our *S. caripense *population using standard methods, we tested chromosome-specific COSII markers that had not only been mapped in tomato, but had amplified fragments in several related solanaceous species and in *Arabidopsis thaliana *[22]. While most of these were non-informative when applied directly, they still contained allelic diversity detectable as SNPs and sufficient to design AS PCR primers.

## Results and discussion

### Amplification and sequencing with non-informative COSII markers

**Figure 1:**
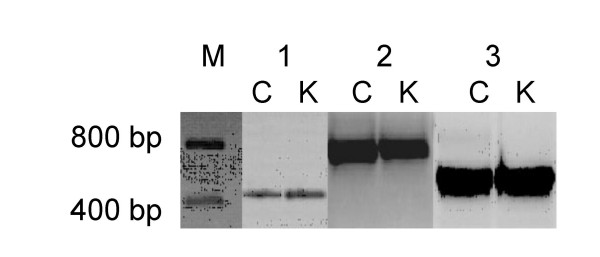
Heterologous PCR fragments visible as single bands of the C and K parents of a *S. caripense *mapping population amplified with COSII markers C2_At5g09880 (1), C2_At5g04590 (2) and C2_At3g54470 (3). The marker (M) indicates fragment sizes.

**Figure 2:**
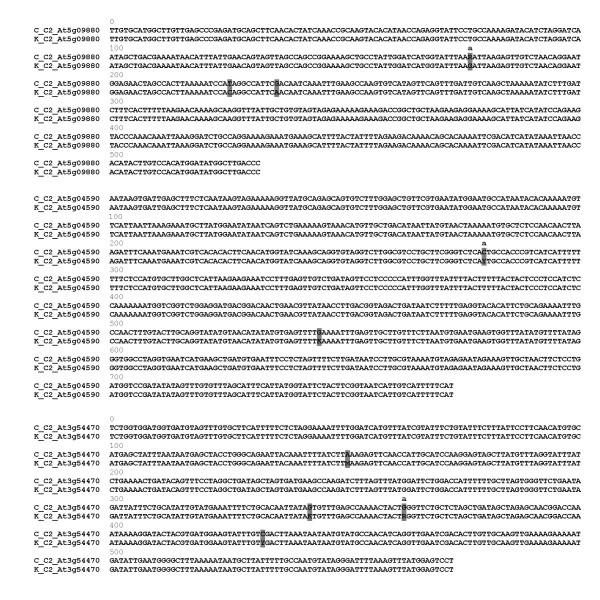
Sequences of PCR fragments amplified from genomic DNA of the C and K parents of the *S. caripense *CK mapping population, for the COSII markers, C2_At5g09880, C2_At5g04590 and C2_At3g54470. SNP positions are shaded. SNPs used for AS SPD primer design are marked with 'a'.

The PCR products obtained from genomic DNA of the parental plants with the three original COSII primer pairs used for illustration here, were non-informative single bands on agarose gels (Fig. 1). Although these PCR fragments appeared visually indistinguishable, direct sequencing revealed several single nucleotide dimorphisms marking the two inherent allele sequences amplified by the COSII primers, as is shown in Fig. 2.

For the design of a selective AS SPD primer, one nucleotide that was dimorphic (indicating heterozygous state) in one parent and monomorphic in the other (homozygous) parent was selected from each COSII marker sequence. For the SPD marker At5g09880-177, the parental genotypes were A/G for parent C and G/G for parent K. Likewise, the C parent carried a C at position 280 of the At5g04590 marker fragment while parent K was heterozygous, C/T. For marker locus At3g54470-363, parent C was homozygous G/G while parent K carried A/G.

**Figure 3:**
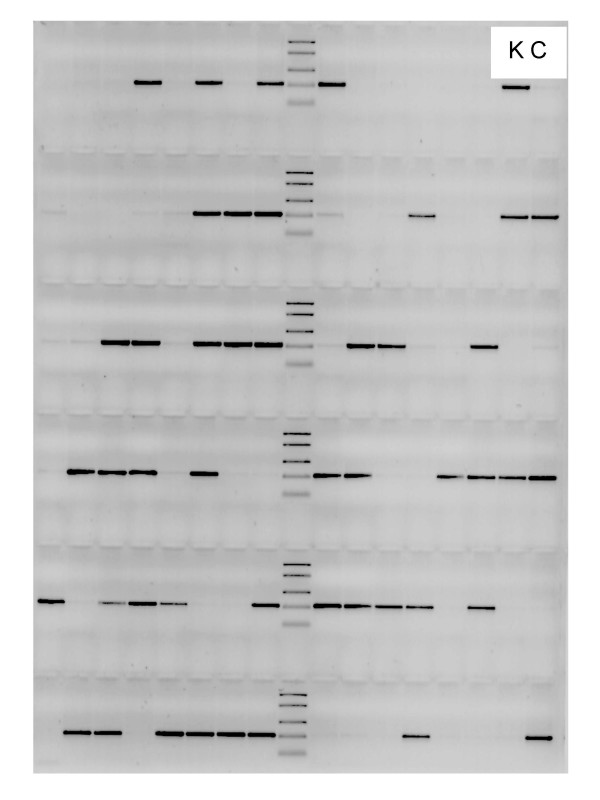
Presence or absence of amplicons following PCR with primers for SPD marker At5g04590-280T originating in parent K (upper right, left lane; parent C, right lane, is devoid of the fragment), in 94 progenies of the CK mapping population revealing the expected 1:1 segregation (46 progenies with, 48 without a fragment).

The AS SPD primers reproducibly amplified a single fragment of the size expected from DNA of plants possessing the corresponding allele. We did not observe any erroneous amplification with mismatched primers on parental and progeny plants whose allelic status was known from sequencing. The SPD markers segregated 1:1 across the entire mapping population, confirming correct detection throughout all DNA samples. Potential primer mismatch was avoided by using limited amounts of magnesium and the higher effective annealing temperatures of LNA-containing primers as compared to pure DNA primers [23,24]. When, for proof of the method, the selective AS SPD primer was replaced with a primer specific for the monomorphic SNP present in the homozygous parent a single fragment was obtained from all plants as expected (data not shown). Among all 186 individuals of the mapping population, SPD marker At5g04590-280T originating from the K parent was present in 92 and absent from 94 progenies corresponding to a 1:1 segregation (partly shown in Fig. 3). Marker At5g09880-177A originating from the C parent occurred in 89 versus 97 progenies and thus also segregated 1:1 as was confirmed by a χ^2^-test for goodness-of-fit (χ^2 ^= 0.344; P>0.55) and marker At3g54470-363T was observed in 88 and absent in 98 individuals (χ^2 ^= 0.538; P>0.46). The 1:1 segregation of all SPD markers confirms the correct functioning of the corresponding AS SPD primers.

We tested the selectivity of our primers containing LNA in real-time PCR and observed 2–6 cycles of signal difference between template from plants containing the specific allele and plants devoid of it. In most cases, a signal was obtained also with plants that do not contain the specific allele targeted by the corresponding primer (not shown). This erroneous amplification may result from the high magnesium concentration in the buffer used (SYBR Green premix, BioRad) which did not create the stringent conditions required for our primers. Reduced levels of magnesium, as applied with the standard PCR (see Methods) should minimize amplification from mismatching primers. The overall discriminatory amplification in real-time PCR confirms results by Maertens *et al*. [25]. The correct functioning of a primer containing terminal LNA nucleotides is also dependent on the specific primer (and template DNA) sequence [17,26]. Therefore, maximum discriminatory power could be achieved with primers containing a combination of a single mismatch nucleotide at the third position from the 3' terminus [27,28] and 3'-terminal LNA or 3'-subterminal ENA (2'-O,4'-C-ethylene nucleoside [29]) [25].

### Genetic mapping of the SPD markers

**Figure 4:**
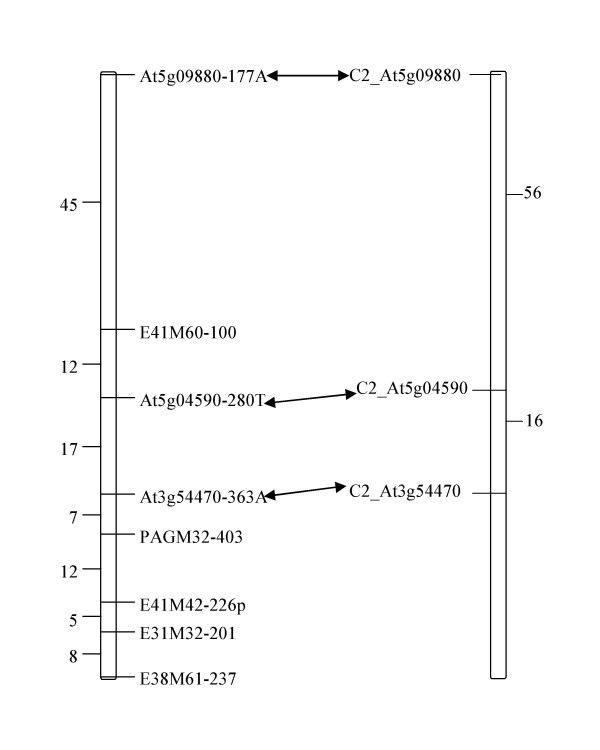
Comparison of genetic distances (centiMorgans) between SPD markers on the *S. caripense *map (left) and markers on the tomato EXPEN 2000 map (right). Arrows connect the original COSII markers and the SPD markers for *S. caripense *developed from them.

The three SPD markers, At5g09880-177A, At5g04590-280T and At3g54470-363A, along with five anonymous AFLP markers mapped to one linkage group on the *S. caripense *map (Fig. 4). The genetic distances between the SPD markers are comparable to those of the original COSII markers on the Tomato EXPEN 2000 map [30], confirming that this linkage group of *S. caripense *corresponds to Solanum chromosome XI.

### Development of a set of chromosome-specific, sequence polymorphism-derived (SPD) markers for *S. caripense*

Having established and proved the principle, the SPD marker protocol was applied to non-informative COSII markers for all 12 Solanum chromosomes in the *S. caripense *mapping population, as is summarised in Table 1. The average length of the COSII marker fragments subjected to sequencing ranged from 149 to 1,382 bp and the average number of SNPs detected per primer pair ranged from none (for 19% of the markers) to 27, with an average of 6 SNPs per marker fragment sequenced. A total of 113 SNPs dimorphic for one *S. caripense *parental genotype and monomorphic for the other, were detected in 37 COSII marker sequences and these were used for genetic mapping. Out of 31 AS SPD primers designed and tested, 23 were successfully used for genotyping the entire population and the results always fitted the expected 1:1 segregation ratio (data not shown). Sixteen marker loci were included in the *S. caripense *genetic linkage map with no difficulty. Seven out of the 12 chromosomes of Solanum were immediately identified in addition to chromosome XI presented above, with genetic distances on the *S. caripense *map comparable to those between the corresponding original COSII markers on the Tomato EXPEN 2000 map.

**Table 1:** SPD markers for chromosomes of *S. caripense*. Summary of number of chromosome-specific COSII primers tested, SNPs detected, and success of genotyping and genetic mapping.

Chromosome (Solanum)	Number of COSII markers tested	Number of SNPs detected	Number of AS SPD primers designed	SPD markers successfully genotyped	SPD markers mapped
I	7	41	4	3	2
II	6	40	3	3	2
III	8	59	2	2	-
IV	7	55	3	2	-
V	6	30	3	2	2
VI	6	17	3	3	3
VII	8	38	2	1	1
VIII	6	47	2	1	1
IX	4	6	2	2	2
X	8	18	3	1	-
XI	8	52	3	3	3
XII	6	64	1	0	-

Total	80	467	31	23	16

The detection of segregating SPD markers and their genetic mapping showed a high success rate in our population of *S. caripense *possessing a highly homogeneous genetic background, as compared to the conventional genotyping methods that had failed (see Background). More COSII- and other markers with known map position in Solanum are being used for amplification, SNP search, and design of SPD markers to assign the four remaining chromosomes.

## Conclusion

Table 2 compares the SPD marker technique to some of the commonly used SNP genotyping techniques, taking into account their accuracy, cost, complexity and equipment required. The SPD protocol is advantageous as it is less time-consuming and requires standard equipment. In fact, once allele-specific SNPs from the parental allele sequences are known, a thermocycler and agarose gel electrophoresis equipment are sufficient for genotyping.

**Table 2:** Comparison of the SPD protocol with other SNP genotyping methods.

Method	Accuracy	Cost	Modification	Complexity	Detection equipment/chemicals	Reference
SPD	High	Low	LNA base at 3' end	Low	Standard^a^	this paper
FRET	High	Medium	Fluorescence	Medium	Optical system	[16]
BAMPER	Medium	High	Bioluminescence	High	PPi assay, Luminometer, Amplifier and Recorder	[28]
PAMSA	Medium	Low	Internal nucleotide mismatch	Medium	Standard	[31]
SNaPshot	High	Very High	Fluorescence	Medium	Sequencer	[32]
AFLP	High	Medium	^33^P or Fluorescence	Medium	Acrylamide gel or sequencer, Restriction enzymes	[33]

^a^Standard; consisting of sequencer, thermocycler and agarose gel electrophoresis equipment, FRET; fluorescence resonance energy transfer, PAMSA; PCR amplification of multiple specific alleles, BAMPER; bioluminometric assay using a modified primer extension reaction, PPi; sodium pyrophosphate decahydrate, AFLP; amplified fragment length polymorphism. Assessment of accuracy, cost, complexity of techniques and data interpretation, and robustness follow the criteria proposed by Kofiadi and Rebrikov [34].

The SPD marker method is not only applicable in population studies but will be very useful in detecting polymorphism among closely related individuals and populations constituted in a narrow genetic background. Though developed and tested in *S. caripense*, the technique can be easily applied in other plants, other conventional marker types and various applications, for example when individuals are closely related to one another. By using AS SPD primers it should be possible to perform SNP genotyping in genetic mapping, genealogical analyses, or gene expression. Favourable attributes of the SPD method include; i) high reproducibility due to the incorporation of a single LNA base at the 3'-end of the selective primer, ii) low cost of marker development; need for sequencing of short fragments only, iii) low cost of application; standard thermocycler and agarose gel electrophoresis equipment is sufficient, and iv) ease of SPD marker detection and reduction of time for marker analysis.

## Methods

### Genetic material

The *S. caripense *CK mapping population generated by crossing two unrelated parents denominated C and K was used. DNA from the parental plants and 186 progenies was extracted following the protocol described by Doyle and Doyle [35].

### PCR amplification using primers for non-informative markers

Three COSII markers previously mapped on tomato chromosome XI (Table 3) were selected from the tomato EXPEN 2000 map [30], for the example used here to illustrate the technique. Parental genomic DNA was amplified by PCR using the selected primers in a 20-μl reaction volume containing 50 ng of genomic DNA, 2 pmol of each primer, 0.2 mM dNTPs (Invitrogen), 1.4 U FirePol polymerase (Solis Biodyne) in 2.0 μl of the buffer supplied with the enzyme and 2.5 mM MgCl_2_. Amplification was done in a GeneAmp PCR System 9700 thermocycler (Applied Biosystems, Forster City, CA, USA) programmed for an initial 1 min at 94°C and 35 cycles of 1 min at 94°C, 1 min at 55°C, and 90 sec at 72°C, followed by a final 10-min extension step at 72°C. Amplification products were visualised by standard 1% agarose gel electrophoresis.

**Table 3:** PCR primers for amplification of COSII markers previously mapped to tomato (*S. lycopersicum*) chromosome XI.

COSII marker	Primer (5'–3')	
	Forward	Reverse

C2_At5g09880	AGGGTCAAGCATATCCATGTGGAC	TCTCTTGTGCATGGCTTGTTGAGC^a^
C2_At5g04590	ATCACCACAGTCCTTGCACAGGG	AGGACAAAGTGGAAAAGCTGGG^a^
C2_At3g54470	TCCTGACTTTGGTTCTAAGCTTAGATCG^a^	TCAAATATTAAGAAGTTGTGCTTGTCTGC

^a^Primers that were also used in combination with the respective AS SPD primer for SNP genotyping (see text; SNP genotyping). Primer source: SOL Genomics Network [30].

### Sequencing and SNP detection

The heterologous fragments amplified from genomic DNA of parents, with COSII primers that appeared as a single band on agarose gels, were cleaned through sephadex (GE Healthcare) on Multiscreen filter plates (Millipore) to remove residual primer. A 10-μl sequencing reaction volume was prepared using 1 μl of the cleaned PCR product, 0.5 μl of either the forward or reverse primer, 1 μl BigDye terminator v3.1 (Applied Biosystems), and 1 μl *half*BD 3.1 (Genetix). The sequencing PCR consisted of 35 cycles of 30 seconds at 94°C, 15 sec at 55°C, and 4 min at 60°C. The sequencing reaction product was cleaned through sephadex columns before adding 10 μl of Hi-Di formamide (Applied Biosystems). The samples were then analyzed on an ABI Prism 3100 Genetic Analyzer automated sequencer (Applied Biosystems) using the default settings. The resulting parental sequences were visually screened for SNPs in Sequencher 4.2 software (GeneCode).

### Design of allele-specific primers

Based on the SNPs detected, allele-specific sequence polymorphism-derived (AS SPD) primers, featuring a single, allele-selective LNA base at their 3'-end were designed using Primer 3 software [36] and purchased from Sigma-Proligo. The LNA (underlined)-containing AS primers were: At5g09880-177A (177 indicates the position of the selective nucleotide, A) with the primer sequence, 5' CTATTGGATCATGGTATTAAA 3', AT5G04590-280T (5' CGTCCTGCTTCGGGTCTCAT 3'), and At3g54470-363A (5' GCTATCAGCTAGAGCAGAACCT 3').

### SNP genotyping with AS SPD markers

The AS SPD primers in combination with the complementary primer of the corresponding original COSII primer pair (Table 3) were used to amplify fragments from genomic DNA of all 186 individuals of the mapping population. The PCR protocol described above for the original COSII primers was used with the modifications; higher annealing temperature (3–5°C increase) and reduced MgCl_2 _concentration (1.8 mM), which further increased the specificity of amplification. Amplicons were detected by electrophoresis in 1%-agarose gels.

### Genotyping with AS SPD markers by real-time PCR

For a total reaction volume of 25 μl, approximately 50 ng of genomic template DNA was mixed with 12.5 μl SYBR Green premix (BioRad) and 320 nM of each primer. Reactions were replicated two times. The RT-PCR was run on an iCycler (BioRad) with 35 cycles of 15 sec at 95°C, 1 min at 61°C, and 1 min at 72°C.

### Genetic mapping of sequence polymorphism-derived (SPD) markers

Polymorphism, indicated by presence vs. absence of a fragment on agarose gels was scored and this SPD marker data was genetically mapped on the parental maps of the CK population using Joinmap 3.0 software [37]. Linkage groups of each parental map were calculated using the Kosambi mapping function with a logarithm of the odds (LOD) score of >3.

## Competing interests

The author(s) declare that they have no competing interests.

## Authors' contributions

JN performed the SNP genotyping, data analysis and genetic mapping, and participated in drafting of the manuscript. FT conceived of the SPD marker method and carried out the real-time PCR. BT developed the CK mapping population of *S. caripense*, conceptualised the project, and participated in drafting of the manuscript. All authors read and approved the final manuscript.
